# The beneficial effects of omega-3 polyunsaturated fatty acids on controlling blood pressure: An umbrella meta-analysis

**DOI:** 10.3389/fnut.2022.985451

**Published:** 2022-08-18

**Authors:** Vali Musazadeh, Zeynab Kavyani, Behnaz Naghshbandi, Parvin Dehghan, Mahdi Vajdi

**Affiliations:** ^1^Student Research Committee, Tabriz University of Medical Sciences, Tabriz, Iran; ^2^Department of Community Nutrition, School of Nutrition and Food Science, Tabriz University of Medical Sciences, Tabriz, Iran; ^3^Department of Food Science and Technology, North Tehran Branch, Islamic Azad University, Tehran, Iran; ^4^Cardiovascular Research Center, Tabriz University of Medical Sciences, Tabriz, Iran; ^5^Student Research Committee, Isfahan University of Medical Sciences, Isfahan, Iran; ^6^Department of Community Nutrition, School of Nutrition and Food Science, Isfahan University of Medical Sciences, Isfahan, Iran

**Keywords:** n-3 PUFAs, blood pressure, systematic review, umbrella of meta-analysis, omega-3 fatty acid

## Abstract

Several meta-analyses have revealed that n-3 PUFAs can lower blood pressure, but the findings are conflicting. In this regard, the present umbrella meta-analysis aimed was performed to clarify whether n-3 PUFAs have effects on blood pressure. PubMed, Scopus, Embase, Web of Science, and Google Scholar were used as international databases from inception to May 2022. To examine the effects of n-3 PUFA supplementation on blood pressure, a random-effects model was applied. The leave-one-out method was performed for the sensitivity analysis. The pooled estimate of 10 meta-analyses with 20 effect sizes revealed significant reductions in both systolic (ES = −1.19 mmHg; 95% CI: −1.76, −0.62, *p* < 0.001) and diastolic blood pressure (ES = −0.91 mmHg, 95% CI: −1.35, −0.47; *p* < 0.001) following n-3 PUFAs supplementation. In studies with a sample size of ≤ 400 participants and a mean age over 45, SBP and DBP were found to be substantially reduced. Overall, this umbrella meta-analysis indicates that n-3 PUFAs supplementation might play a role in improving DBP and SBP.

## Introduction

Hypertension is among the most common health conditions worldwide affecting over 1 billion people and accounting for 9.4 million deaths annually (1). Elevated blood pressure is responsible for heart attacks, arrhythmias, stroke and premature death (2). Research shows that some recommendations such as healthy dietary habits, and smoking cessation can reduce blood pressure (BP), thereby decreasing hypertension and CVD risks (3).

Omega-3 polyunsaturated fatty acids (PUFAs) mainly consist of docosahexaenoic acid (DHA), eicosapentaenoic acid (EPA) and alpha-linolenic acid (ALA) (4). Diets deficient in n-3 PUFAs from seafood were classified as the sixth most significant dietary risk factor −1.5 million deaths and 33 million disability-adjusted life years worldwide were attributed to this (5). According to most national and international guidelines, adults should consume at least one serving of oily fish per week (≥ 250 mg/day of EPA and DHA) (6, 7). Despite this, an estimated 20% of the world’s population consumes less than 250 mg of EPA and DHA per day (8). Although the role of PUFAs in hypertension is still subject to an scientific debate, they have been shown to have multiple beneficial effects in cardiovascular disease (9). Additionally, they demonstrated that they had anti-inflammatory (10) and antithrombotic (11) properties, improved endothelial dysfunction (12) and positively affected resting heart rate (HR), HR variability (13, 14), heart rhythm (15), and cardiac remodeling (16). In terms of their antihypertensive properties, n-3 PUFAs were reported to modulate ion channels in blood vessels, thereby causing vasodilation (17). The red blood cell membrane omega-3 content of humans is associated with smaller brachial artery diameter (an independent predictor of cardiovascular events) and increased vasodilatory function (18). Omega-3 content in red blood cell membranes of humans is associated with smaller brachial arteries (an indicator of cardiovascular events) and increased vasodilation (18). Generally, n-3 PUFAs were shown to improve arterial compliance and reduce pulse wave velocity. Some benefits of omega-3-PUFAs may be mediated through downstream effects on BP (8, 19, 20). According to a report by Averna et al. (21) high-dose omega-3 fatty acids (Icosapent ethyl) may be considered a benefit to managing TG-rich lipoproteins. By TG-lowering, the risk of cardiovascular events decreases. In recent years, several systematic reviews and meta-analyses of randomized controlled trials have been performed investigating the effects of n-3 PUFAs supplementation on BP (22, 23), while In some studies, controversial results have been reported (24–26). Therefore, the present umbrella meta-analysis was performed to assess the explicit impact of n-3 PUFA supplementation on systolic BP (SBP) and diastolic BP (DBP) providing valid and authentic evidence.

## Methods

The current umbrella of meta-analysis was performed based on the PRISMA. We registered our study protocol in PROSPERO (CRD42022311888).

### Search strategy of literature

Searches were conducted in PubMed, Scopus, Embase, Web of Science, and Google Scholar databases to find relevant studies published inception to May 2022 The following search strategy using MeSH terms and keywords was applied; [“omega-3 fatty acid” (Mesh) OR “omega-3 fatty acid” (tiab) OR “fish oil” (tiab) OR “omega-3 polyunsaturated fatty acids” (tiab) OR “EPA” (Mesh) OR “DHA” (Mesh) OR “DHA” (tiab) OR “EPA” (tiab) **AND** “hypertension” (Mesh) OR “hypertension” (tiab) OR “HTN” (tiab) OR “blood pressure” (tiab) OR “BP” (tiab) OR “hypertens” (tiab) **AND** “systematic review” (Publication Type) OR “meta-analysis” (tiab)]. Additionally, we enhanced sensitivity of the search strategy by using the wildcard term “*”. To ensure no publications were missing, reference lists of relevant studies were manually screened. In this umbrella of meta-analysis, English-language articles were eligible.

### Inclusion and exclusion criteria

A PICO analysis was conducted to summarize the results of the current umbrella meta-analysis. The PICO criteria were: population/patients (P: adults over 18 years’ old who received omega-3 PUFAs); intervention (I: omega-3 PUFAs therapy); comparison (C: control or placebo group); outcomes (O: BP including SBP and DBP). Our study included meta-analyses that assessed the effects of n-3 PUFA supplementation on BP (SBP and DBP) along with their effect sizes (ES) and corresponding confidence intervals (CI). *In vivo*, *in vitro*, and *ex vivo* studies, observational studies, quasi-experimental studies, case reports, and controlled clinical trials were excluded.

### Evaluation of methodological quality

In terms of their methodology, two independent reviewers (VM and MV) performed AMSTAR2 (Assessing the Methodological Quality of Systematic Reviews) questionnaires to evaluate the quality of meta-analyses (27). The questionnaire consists of 16 questions that ask reviewers to answer “Yes” or “Partial Yes” or “No” or “No Meta-analysis.” Four categories were developed for the AMSTAR 2 checklist: “Critical low quality,” “low quality,” “moderate quality,” and “high quality.

### Study selection and data extraction

Based on eligibility criteria, articles were screened by two independent reviewers (MV and VM). After reviewing based on their titles and abstracts, irrelevant studies were removed. Then, by evaluating the full text of the relevant articles, eligible studies for the umbrella of meta-analysis were identified. Finally, any disagreement was resolved by discussion and consulting with other researchers (PD). The first author’s name, the publication year, the sample size, the study location, supplementation dosages, and duration, as well as the ESs and CIs for SBP and DBP, are extracted from the selected meta-analyses.

### Synthesis of data and statistical analysis

ESs and CIs were applied to estimate overall effect sizes. Cochran’s *Q* test and *I*^2^ statistics were used to determine heterogeneity. When *I*^2^-value > 50% or the *Q*-test had *p* < 0.1, we considered between-study heterogeneity significant. The random-effects model was chosen when there was significant between-study heterogeneity. By performing subgroup analysis based on predefined variables including sample size, duration of intervention, health condition, the dose of supplementation, and mean age, potential sources of heterogeneity were detected. Sensitivity analysis was conducted to investigate the impact of one single study omission on the pooled effect size. Tests Egger’s and Begg’s were used to assessing a small-study effect. The publication bias was assessed by visual inspection of the funnel plots, and if any publication bias was detected, then a trim and fill test was carried out subsequently. In order to run the meta-analysis, we used STATA (version 16, Stata Corporation, College Station, TX, United States). In this study, *p* < 0.05 was considered statistically significant.

## Results

### Study selection and study characteristics

**FIGURE 1:**
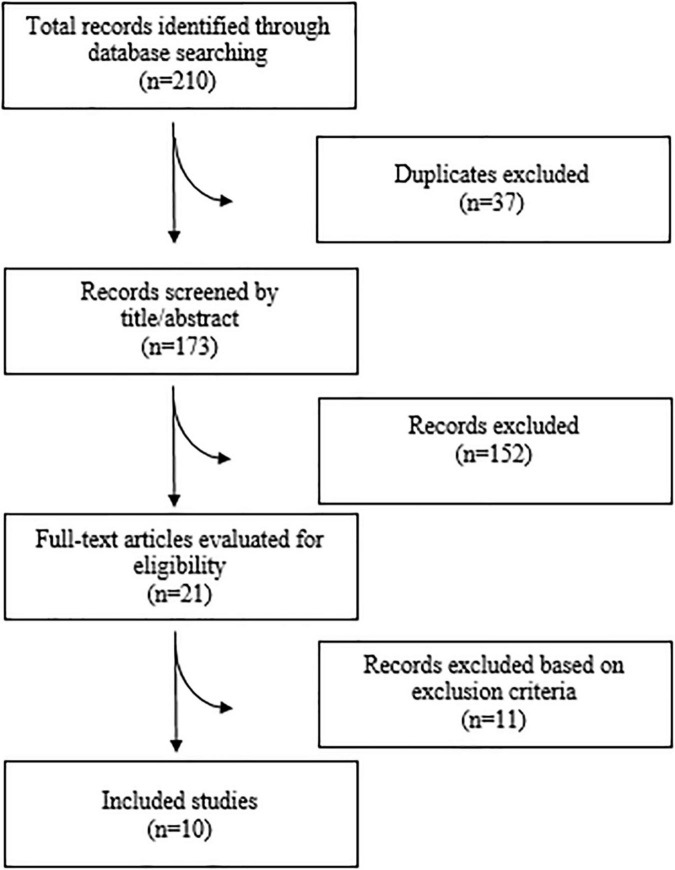
Flow diagram of study selection.

A total of 210 articles were found after searching the electronic databases. 173 articles were carefully screened with titles and abstracts, after removing 37 duplicate articles, of which 21 articles were selected for full-text evaluation. Finally, 10 meta-analyses were included in the current umbrella meta-analysis. Figure 1 illustrates the flow chart of PRISMA studies. The characteristics of the meta-analyses that were qualified are summarized in Table 1. All studies were carried out between 1989 and 2021, and the participants ranged in age from 34 to 55 years. There was an average amount of 2.2–6 g/day of administered n-3 PUFAs across studies. The duration of n-3 PUFA supplementation ranged from 4 to 29 weeks. The location of the studies performed were as follows: three in United States (25, 28, 29), two in China (24, 30), two in United Kingdom (22, 31), two in Canada (23, 26), and one in Australia (32). The quality of the randomized controlled trials (RCTs) included in the current study is presented in Table 1.

**TABLE 1:** Study characteristics of included studies.

References	No. of studies in meta-analysis	Location duration	No. of participants in meta-analysis	Age (year)	Health condition	Dose (mg) EPA	Dose (mg) DHA	Dose (mg) EPA + DHA	Quality assessment scale and outcome
Zhang et al. (24)	13	China 16.5 W	1023	53.5	MetS	2314	2100	–	Yes (Jadad) 7/13 high
Lim et al. (32)	3	Australia 7 W	206	NR	Kidney Transplant Recipients	13500	–	6000	Yes (Cochrane) 1/3 high
Campbell et al. (22)	17	UK 12.5 W	475	52	HTN	2,315	1,100	3,457	Yes (Cochrane) 15/17 high
Chewcharat et al. (25)	11	Thailand 25 W	376	53	DN	–	–	2,250	Yes (Cochrane) 5/11 high
O’Mahoney et al. (31)	13	UK 20 W	681	NR	T2DM	1,526	1,568	–	Yes (Cochrane) 13/13 high
Radack and Deck (29)	4	USA 4 W	148	34	Hypertension/Normotensive	–	–	5,725	Yes (Cochrane) 3/4 high
Guo et al. (30)	20	China 11 W	776	55	Dyslipidemia, T2DM, healthy	2,550	2,333	–	Yes (Jadad) 12/20 high
Appel et al. (23)	17	Canada 10 W	1,039	39	Hypertension/normotensive	2,916	2,073	5,177	Yes (Cochrane) 15/17 high
Morris et al. (28)	31	United States 7 W	1,458	46	Hypercholesterolemia, healthy, CVD, HTN, T2DM,	–	–	4,524	No
Musa-Veloso et al. (26)	3	Canada 29 W	189	43	NAFLD	1,216	1,425	–	Yes (Jadad) 3/3 high

NR, Not reported; T2DM, type 2 diabetes mellitus; DN, Diabetic Nephropathy; CVD, Cardiovascular disease; HTN, Hypertension; NAFLD, Non-Alcoholic Fatty Liver; MetS, metabolic syndrome.

### Methodological quality

Results of the quality assessment of the qualified studies using the AMSTAR2 questionnaire are presented in Table 2.

**TABLE 2:** Assessments of the methodological quality of included studies using the AMSTAR2 checklist.

Study	Q1*	Q2	Q3	Q4	Q5	Q6	Q7	Q8	Q9	Q10	Q11	Q12	Q13	Q14	Q15	Q16	Quality assessment
O’Mahoney et al. (31)	No	Yes	Yes	Partial Yes	Yes	Yes	Yes	Yes	Yes	Yes	Yes	Yes	Yes	Yes	Yes	Yes	High
Chewcharat et al. (25)	No	Partial Yes	Yes	Yes	Yes	Yes	Partial Yes	Yes	Yes	No	Yes	Yes	Yes	Yes	No	No	Moderate
Zhang et al. (24)	No	Partial Yes	Yes	Yes	Yes	Yes	Partial Yes	Yes	Yes	Yes	Yes	Yes	Yes	Yes	Yes	Yes	High
Lim et al. (32)	No	Partial Yes	Yes	Partial Yes	No	Yes	No	Yes	No	No	Yes	Yes	Yes	No	No	No	Low
Guo et al. (30)	No	Yes	Yes	Partial Yes	Yes	Yes	No	Partial Yes	Yes	Yes	Yes	Yes	Yes	Yes	Yes	Yes	High
Musa-Veloso et al. (26)	Yes	Yes	Yes	Partial Yes	Yes	Yes	Yes	Partial Yes	Yes	Yes	Yes	Yes	Yes	Yes	Yes	Yes	High
Campbell et al. (22)	No	Partial Yes	Yes	Partial Yes	Yes	Yes	Yes	Yes	Yes	No	Yes	Yes	Yes	No	No	Yes	Moderate
Radack and Deck (29)	No	Partial Yes	No	Partial Yes	Yes	Yes	No	Yes	Yes	No	Yes	Yes	No	No	No	No	Low
Appel et al. (23)	No	Partial Yes	Yes	Partial Yes	No	Yes	No	Partial Yes	Yes	No	Yes	No	No	No	No	No	Critically low
Morris et al. (28)	No	Yes	No	Partial Yes	No	Yes	No	Partial Yes	Yes	No	Yes	No	No	No	No	No	Critically low

*1. Did the research questions and inclusion criteria for the review include the components of PICO? 2. Did the report of the review contain an explicit statement that the review methods were established prior to the conduct of the review and did the report justify any significant deviations from the protocol? 3. Did the review authors explain their selection of the study designs for inclusion in the review? 4. Did the review authors use a comprehensive literature search strategy? 5. Did the review authors perform study selection in duplicate? 6. Did the review authors perform data extraction in duplicate? 7. Did the review authors provide a list of excluded studies and justify the exclusions? 8. Did the review authors describe the included studies in adequate detail? 9. Did the review authors use a satisfactory technique for assessing the risk of bias (RoB) in individual studies that were included in the review? 10. Did the review authors report on the sources of funding for the studies included in the review? 11. If meta-analysis was performed, did the review authors use appropriate methods for statistical combination of results? 12. If meta-analysis was performed, did the review authors assess the potential impact of RoB in individual studies on the results of the meta-analysis or other evidence synthesis? 13. Did the review authors account for RoB in individual studies when interpreting/discussing the results of the review? 14. Did the review authors provide a satisfactory explanation for, and discussion of, any heterogeneity observed in the results of the review? 15. If they performed quantitative synthesis, did the review authors carry out an adequate investigation of publication bias (small study bias) and discuss its likely impact on the results of the review? 16. Did the review authors report any potential sources of conflict of interest, including any funding they received for conducting the review?

Each question was answered with “Yes”, “Partial Yes” or “No”. When no meta-analysis was done, question 11,12 and 15 were answered with “No” meta-analysis conducted.

### Impact of n-3 PUFAs on diastolic blood pressure

**FIGURE 2:**
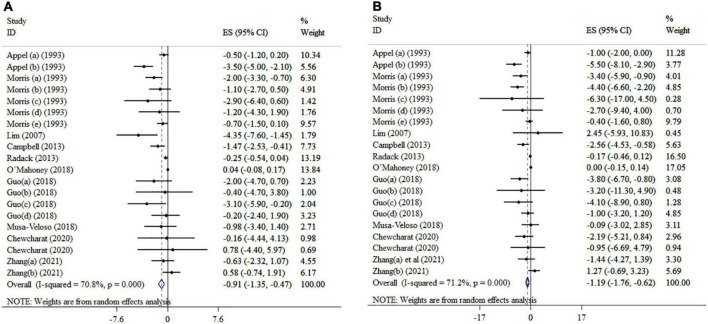
Mean difference and 95% CIs presented in forest plots of the studies on the effects of n-3 PUFA supplementation on DBP **(A)**, and SBP **(B)** levels (a, b, c, d, etc. indicates a separate effect size for different diseases in each study).

Overall, ten included meta-analyses with 20 effect sizes comprising a total of 6,334 subjects have examined the impact of n-3 PUFAs supplementation on DBP (Figure 2A). It was found that DBP was significantly reduced after n-3 PUFAs supplementation (ES = −0.91 mmHg, 95% CI: −1.35, −0.47; *p* < 0.001), with high heterogeneity between-studies (*I*^2^ = 70.8%, *p* < 0.001). N-3 PUFA supplementation > 4,500 mg/day to hypertension subjects > 45 years of age and a sample size of ≤ 400 significantly reduced DBP (Table 3).

**TABLE 3:** Subgroup analyses for the effects of omega-3 fatty acids supplementation on blood pressure.

	Effect size, *n*	ES (95% CI)^a^	*P*-within^b^	*I*^2^ (%)^c^	*P*-heterogeneity^d^
**Omega-3 fatty acids supplementation on DBP**					
Overall	20	−0.91 (−1.53,−0.47)	<0.001	70.8	<0.001
**Age (year)**					
≤45	7	−0.36 (-0.61,−0.11)	0.005	0.0	0.873
>45	11	-1.43 (-2.33,−0.53)	**0.003**	55.8	0.012
NR	2	−1.88 (-6.14, 2.39)	0.389	87.2	0.005
**Intervention duration (week)**					
≤10	12	−0.81 (-1.34,−0.27)	0.003	50.6	0.022
10–15	2	−2.42 (-4.41,−0.44)	0.017	79.6	0.027
>15	6	0.03 (-0.09, 0.15)	0.641	0.0	0.616
**Sample size**					
≤400	13	−1.55 (-2.52,−0.57)	0.002	63.3	<0.001
>400	7	−0.60 (-1.18,−0.01)	0.047	73.7	<0.001
**Study population**					
T2DM	3	−0.04 (-0.09, 0.16)	0.345	0.0	0.721
HTN	4	−1.70 (−3.12, −0.27)	0.020	89.1	0.040
DN	2	−0.22 (−3.08, 3.52)	0.895	0.0	0.784
NAFLD	1	−0.98 (−3.38, 1.42)	0.555	–	–
Kidney transplant recipients	1	−4.35 (−7.42, −1.27)	0.030	96.5	<0.001
Metabolic disorders	4	−0.55 (−1.69, 0.59)	0.012	38.4	0.182
Healthy	3	−0.56 (−1.08, −0.05)	0.424	0.0	0.881
Dyslipidemia	2	−2.52 (−4.48, −0.56)	0.006	0.0	0.583
**EPA (mg/day)**					
≤2,000	4	−0.02 (−0.10, 0.14)	0.736	0.0	0.397
>2,000	7	−1.95 (−3.95, −0.86)	<0.001	45.6	0.088
**DHA (mg/day)**					
≤1,700	6	−0.44 (−1.04, 0.16)	0.149	52.4	0.062
>1,700	4	−1.61 (−4.18, 0.96)	0.219	83.6	<0.001
**EPA + DHA (mg/day)**					
≤4,500	5	−0.80 (−1.25, −0.35)	<0.001	0.0	0.645
>4,500	5	−2.34 (−4.13, −0.56)	0.010	87.6	<0.001
**Omega-3 fatty acids supplementation on SBP**					
Overall	20	−1.19 (−1.76, −0.62)	<0.001	72.1	<0.001
**Age (year)**					
≤45	8	−1.05 (−1.92, −0.18)	0.018	61.9	0.010
>45	10	−**2.60 (**−**4.22,** −**0.97)**	0.002	59.1	0.009
NR	2	0.00 (−0.14, 0.15)	0.992	0.0	0.567
**Intervention duration (week)**					
≤10	12	−1.16 (−2.09, −0.24)	0.013	62.9	0.002
10–15	3	−2.48 (−5.64, 0.68)	0.106	91.5	<0.001
>15	5	−**1.81 (**−**3.23,** −**0.39)**	0.116	0.0	0.512
**Sample size**					
≤400	11	−**2.39 (**−**4.23,** −**0.55)**	0.011	72.2	<0.001
>400	9	−1.01 (−1.86, −0.15)	0.021	70.3	<0.001
**Study population**					
MetS	4	−1.87 (−5.21, 1.48)	0.275	80.1	0.002
T2DM	3	−0 (−0.15, 0.14)	0.975	0	0.543
HTN	4	−**2.71 (**−**5.26,** −**0.17)**	0.037	88.9	<0.001
DN	2	−1.92 (−4.60, 0.76)	0.160	0	0.708
Dyslipidemia	2	−3.88 (−6.40, −1.36)	0.003	0	0.918
Healthy	3	−0.78 (−1.51, −0.06)	0.035	0	0.737
Kidney transplant recipients	1	2.45 (−5.93, 10.83)	0.567	−	−
NAFLD	1	−0.09 (−3.02, 2.85)	0.953	–	–
**EPA (mg/day)**					
≤2,000	4	−0.23 (−0.78, 0.32)	0.414	22.4	0.276
>2,000	7	−**2.98 (**−**4.25,** −**1.70)**	<0.001	15.4	0.312
**DHA (mg/day)**					
≤1,700	5	−0.74 (−1.72, 0.25)	0.143	60.7	0.038
>1,700	5	−2.08 (−4.66, 0.50)	0.114	78.1	<0.001
**EPA + DHA (mg/day)**					
≤4,500	5	−1.85 (−3.22, −0.47)	0.009	66.3	0.018
>4,500	5	−2.55 (−5.65, 0.54)	0.106	83.1	<0.001

ES, Effect size; CI, confidence interval; NR, Not reported; T2DM, type 2 diabetes mellitus; DN, Diabetic Nephropathy; CVD, Cardiovascular disease; HTN, Hypertension; NAFLD, Non-Alcoholic Fatty Liver; MetS, metabolic syndrome.

^a^Obtained from the Random-effects model.

^b^Refers to the mean (95% CI).

^c^Inconsistency, percentage of variation across studies due to heterogeneity.

^d^Obtained from the Q-test.

### Impact of n-3 PUFAs on systolic blood pressure

The effect of n-3 PUFAs supplementation on SBP was investigated in ten meta-analyses with 20 effect sizes, including 6,734 subjects. The pooled estimate revealed that in subjects who consumed n-3 PUFAs supplements, SBP significantly was reduced (ES = −1.19 mmHg; 95% CI: −1.76, −0.62, *p* < 0.001) (Figure 2B). There was detected meaningful heterogeneity between the studies (*I*^2^ = 71.2%, *p* < 0.001). Subgroup analysis showed that EPA supplementation (> 2,000 mg/day) in intervention duration of > 15-weeks, a mean age > 45 years, and a sample size of ≤ 400 participants contributes to a more pronounced influence in decreasing SBP (Table 3).

### Sensitivity analysis and publication bias

Sensitivity analysis indicated that the effect of n-3 PUFAs supplementation on DBP and SBP levels was not significant (Supplementary File 1).

**FIGURE 3:**
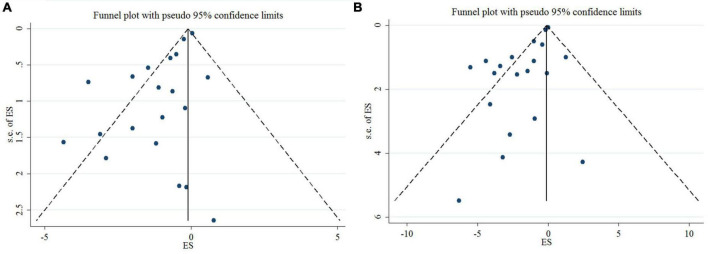
Mean difference and 95% CIs presented in funnel plots; and publication bias of the studies the effects of n-3 PUFA supplementation on DBP **(A)**, and SBP **(B)** levels.

Egger’s test, unlike Begg’s, showed a significant small-study effect for DBP (*p* = < 0.001 and 0.626, respectively), and SBP (*p* = < 0.001 and 0.948, respectively). In addition, publication bias was detected through a visual assessment of the funnel plots (Figure 3). Trim and fill adjustment did not alter the overall effect size.

## Discussion

The current umbrella meta-analysis of the n-3 PUFAs effect on BP indices summarized 10 meta-analyses including 131 trials. Although several studies have evaluated the effect of n-3 PUFAs on CVD risk factors, inconsistent findings have been achieved in terms of the effects of n-3 PUFAs on the BP because significant changes have not been observed consistently. Hence, we performed the present study to systematically assess the treatment effects of n-3 PUFAs on the BP in adults. Our results revealed that n-3 PUFAs supplementation can significantly decrease SBP, and DBP levels, with moderate confidence of evidence. Nevertheless, there was significant heterogeneity in the response which attributed to subject age, study population, study duration, sample size, and dosage of n-3 PUFAs. Moreover, we also perform subgroup analysis to gain further insight on the assessment of heterogeneity. Subgroup analysis revealed the effects of n-3 PUFAs were stronger in hypertensive patients, and among studies with a follow-up period of at least 10 weeks. Also, DBP and SBP were both reduced in studies lasting up to 10 weeks with daily dose of EPA > 2,000 mg/day. The quality of studies was assessed in all meta-analyses included in our study except for Morris et al. (28). Except three studies that used Jadad scale, other meta-analyses used Cochrane Collaboration’s tool to evaluate the different sources of bias in trials. Additionally, the majority of the included articles were of high risk of bias based on quality assessment.

High BP is a modifiable risk factor that can lead to cardiovascular disease (CVD), stroke, renal disease, and mortality (33, 34). In recent decades, several publications have showed that therapy with n-3 PUFAs positively effect on BP (22, 30, 35). Actually, several previous studies have reported that n-3 PUFAs may decrease CVDs risk factors by improving the inflammation (31), lipid, and glycemic profile (24, 36, 37), as well as decreasing BMI (38). In addition to above factors, the effects of n-3 PUFAs supplementation on apolipoproteins, particularly Apo-CIII has been found in recent meta-analysis (39). The findings of our study show that supplementation with n-3 PUFAs decreases DBP as well as SBP. While the magnitude of the detected effect seems small, it appears to be both clinically and statistically significant. According to previous meta-analysis, even a slight reduction in BP is helpful for CVD risk management and can efficiently decrease CVD events (40). A meta-analysis has reported that every 1 mmHg decrease in SBP leads to a nearly 2.5% sex- and age -specific reduction in vascular mortality (41). Consequently, n-3 PUFAs can be considered as an effective and without life-threatening side effects agent in control of BP.

Our results are consistent with three other meta-analyses (22, 30, 35). In the analysis of DBP, subgroup analyses indicated a stronger effect in patients with dyslipidemia, and hypertension, as well as in kidney transplant recipients. A BP lowering effect has been detected in both hypertensive and normotensive subjects, but the response appears more pronounced among hypertensive patients. Furthermore, supplementation of > 2,000 mg/d of EPA appears to be sufficient to reduce both DBP and SBP by 1.95 and 2.98 mmHg, respectively. Stratifying the meta-analysis based on the mean age of subjects revealed that n-3 PUFAs among participants with > 45 years had the most improving effect on SBP and DBP levels compared with younger participants. Long-term supplementation of n-3 PUFAs (10–15 week) led to a larger decrease in DBP levels than ≤ 10-week administration. However, given the findings of our subgroup analysis, it appears that short-term supplementation (≤ 10-week) with n-3 PUFAs, is more effective in decreasing SBP than long-term one. It should also be noted that most included meta-analysis in our review had participants with metabolic disorders, hypertension, and T2DM. Cornelissen and Fagard (42) have shown that hypertensive and normotensive participants may respond in different degrees to the same intervention. Hypertensive patients appear to be a better target population to study the hypotensive effect of n-3 PUFAs because they are more prone to changes in BP (43).

N-3 PUFAs may exert its hypotensive effects by several mechanisms. N-3 PUFAs may control the caveolae composition resulting in increased nitric oxide synthase (44). In particular, n-3 PUFAs stimulate expression and activity of endothelial nitric oxide synthase (45). The BP lowering effect of n-3 PUFAs might be ascribed to enhancements of endothelial and smooth muscle function together with lowered systemic vascular resistance (46). Also, n-3 PUFAs acts as a hypotensive agent through increasing synthesis of vasodilator mediators such as prostacyclin, and inhibiting vasoconstrictor mediators such as thromboxane which are associated with hypertension (47). Prostacyclin exerts its vasodilatory effects by activating prostacyclin receptors present on vascular smooth muscle cells and platelets, which therefore decreases peripheral vascular resistance and arterial stiffness (48). Furthermore, hypotensive property of n-3 PUFAs may be the result of its ability to significantly reduction of TC and LDL-C (31, 49). In a meta-analysis by Zhang et al. (24) significant reduction in serum TC level was observed following EPA supplementation 2 g/day. N-3 PUFAs supplementation possesses several antioxidant properties, combats intracellular reactive oxygen species, and can enhance other antioxidant defense, such as thioredoxin reductase 1, heme oxygenase-, and manganese superoxide dismutase, thus protecting endothelial cells from oxidative functional damages and regulating BP (50, 51). It has also been reported that n-3 PUFAs can counteract the release of pro-inflammatory cytokines in the myocardium and vascular endothelium, so restoring vascular reactivity and myocardial performance (50). Hence, the anti-inflammatory actions of n-3 PUFAs may be another putative way by which antihypertensive effects are exerted.

The main strength of this review was that this umbrella meta-analysis provided a comprehensive summary of the literature and its results and given the potential effect on the management and treatment of hypertension, this is a useful attempt. We revealed that there is sufficient evidence for n-3 PUFAs to elicit helpful effects on blood pressure. In the sensitivity analysis, no changes in the results were observed excluding each meta-analysis; this supports the robustness of our results. However, current study has several limitations. The analyses involved subjects with several health statuses such as T2DM, dyslipidemia, kidney transplant recipients, NAFLD, and metabolic syndrome. While this allowed more meta-analyses and subjects to be included for analysis, this could affect generalizability. However, we done random effects model in our analyses as well as explored for heterogeneity, the current findings are limited by possible residual confounding such as different background diets of participants. Finally, the proportion of n-6 PUFAs: n-3 PUFAs may play an important role in decreasing the risk factors of CVDs (52–54). However, we did not recognize any meta-analysis to involve the relationship between the ratio of n-3/n-6 PUFAs and BP.

## Conclusion

In summary, this umbrella meta-analysis of 10 meta-analyses revealed that, compared with control, n-3 PUFAs intake was related to meaningfully greater degree of SBP, and DBP decrease, and this effect was more obvious in hypertensive subjects. Consequently, supplementation with n-3 PUFAs may be a useful adjuvant therapy in hypertensive patients.

## Data availability statement

The original contributions presented in this study are included in the article/Supplementary material, further inquiries can be directed to the corresponding authors. All the materials used in this systematic review and meta-analysis have been fully referenced.

## Author contributions

VM and PD designed research. ZK and MV conducted a systematic search. BN and ZK screened articles. ZK and VM extracted data and drew tables. VM and MV analyzed and interpreted the data. MV, VM, and ZK wrote the manuscript. PD had primary responsibility for the final content. All authors read and approved the final manuscript.
