# Supplementary figures and images for: The beneficial effects of omega-3 polyunsaturated fatty acids on controlling blood pressure: An umbrella meta-analysis
Source: Front Nutr. 2022 Aug 18;9:985451. doi: 10.3389/fnut.2022.985451 (PMC9435313; doi:10.3389/fnut.2022.985451)

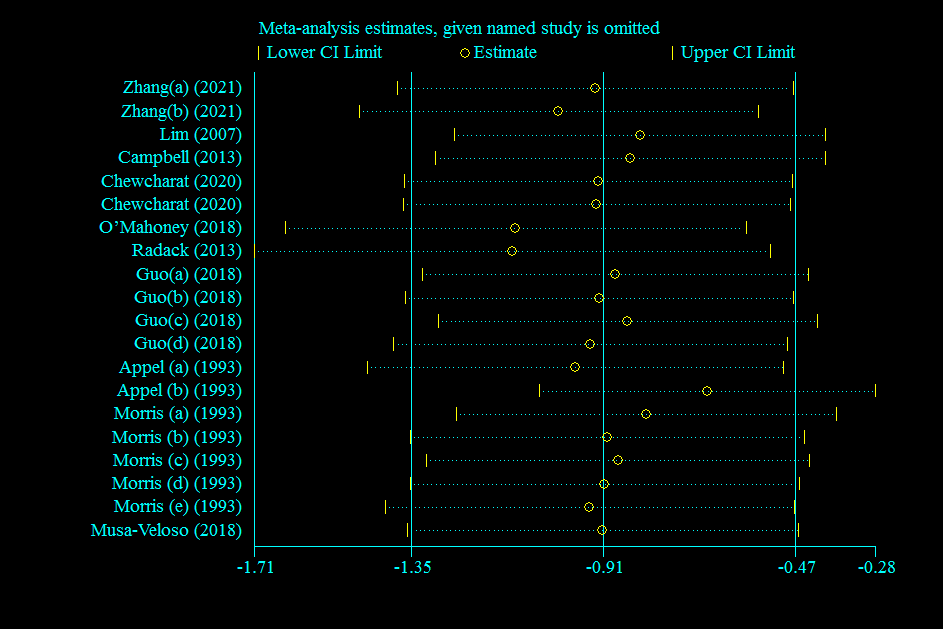


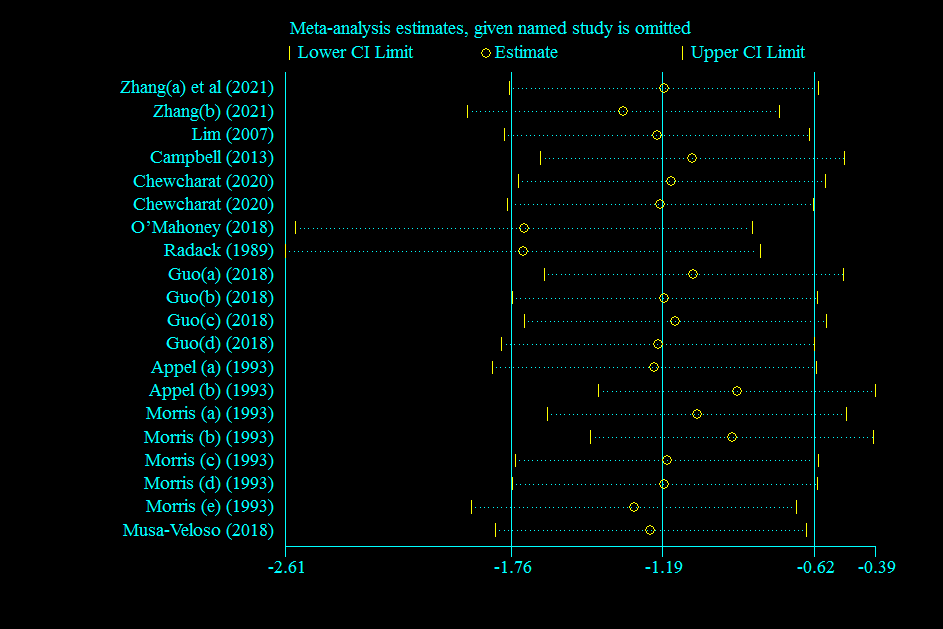


**A**: DBP, **B**: SBP

Supplement: Supplementary file 1 [file Table_1.DOCX]
